# Impact of COVID-19 Pandemic on Bronchiolitis Epidemiology in Greece

**DOI:** 10.3390/medicina61101746

**Published:** 2025-09-25

**Authors:** Athina Koloi, Dimitra Dimopoulou, Dimitris Papakonstantinou, Georgios Damianos, Vasiliki Korentzelou, Marina Triantafyllia Kotzamani, Ariadni Neofytou, Christos Paraschos, Sofia D. Pasparaki, Agori Rizargioti, Kiriaki Benetatou, Maria Tampouratzi, Anastasia Konidari, Alexandra Soldatou, Maria N. Tsolia

**Affiliations:** 1 Second Department of Pediatrics, School of Medicine, “P. and A. Kyriakou” Children’s Hospital, National and Kapodistrian University of Athens, 11527 Athens, Greece; athina_koloi@outlook.com (A.K.); gdamianos1135@gmail.com (G.D.); kotza.mari.tria.94@gmail.com (M.T.K.); arianeophytou@gmail.com (A.N.); spasparaki97@gmail.com (S.D.P.); kiriakiben@yahoo.gr (K.B.); mania.tabouratzi@gmail.com (M.T.); alex_soldatou@hotmail.com (A.S.); mariantsolia@gmail.com (M.N.T.); 2 Third Department of Surgery, School of Medicine, Attikon General University Hospital of Athens, National and Kapodistrian University of Athens, 12462 Athens, Greece; dimpapa7@hotmail.com; 3 First Department of Pediatrics, “P. and A. Kyriakou” Children’s Hospital, 11527 Athens, Greece; vkorentzelou@gmail.com; 4 Second Department of Pediatrics, “P. and A. Kyriakou” Children’s Hospital, 11527 Athens, Greece; chrisparaschos@yahoo.com (C.P.); rea.rizargia@gmail.com (A.R.); anastasia.konidari@gmail.com (A.K.)

**Keywords:** bronchiolitis, epidemiology, Greece, respiratory syncytial virus (RSV), COVID-19 pandemic, severity

## Abstract

*Background and Objectives:* Acute bronchiolitis is a leading cause of hospitalization and morbidity in infants and young children. The COVID-19 pandemic has impacted its epidemiology globally. This study aims to assess changes in bronchiolitis epidemiology in Greece during the period of COVID-19 restrictive measures and after their removal, compared to the pre-pandemic period. *Materials and Methods*: A retrospective non-interventional study was conducted at a tertiary pediatric hospital, including all children aged 0–24 months hospitalized for bronchiolitis between 1 November 2017 and 30 September 2024. A total of 1505 cases were included. Data were analyzed across seven seasonal periods. *Results:* Bronchiolitis hospitalizations decreased by 98% in 2020–2021, returned to pre-pandemic levels in 2021–2022, and increased by 58.7% in January 2022–2023. The seasonal distribution shifted earlier in 2021–2022, with a return to pre-pandemic patterns in 2022–2023 and 2023–2024. No shift was observed in the peak age group (1–3 months), although neonatal hospitalizations decreased significantly in 2020–2021 (*p* = 0.009). RSV-positive cases dropped notably during 2020–2021 (41.2%). There was no change in disease severity across periods, assessed by indirect indicators and the Modified Tal Score. The proportion of bronchiolitis cases among total pediatric admissions increased to 5.6% in 2023–2024, compared to 4.9% in 2022–2023 and 3.9% pre-pandemic (2017–2020). *Conclusions:* Bronchiolitis epidemiology was significantly impacted by the COVID-19 pandemic in Greece, though disease severity remained unchanged. Ongoing monitoring of bronchiolitis and RSV circulation is crucial particularly with the introduction of maternal RSV vaccination and new monoclonal antibodies to inform prevention strategies and reduce disease burden.

## 1. Introduction

The COVID-19 pandemic posed a serious threat to public health, resulting in increased morbidity and mortality globally [1]. Due to the rapid spread of the virus and the absence of population vaccination, public health authorities recommended measures to limit the transmission of the infection, such as social distancing and personal protective measures [2]. Before the emergence of the COVID-19 pandemic, bronchiolitis was one of the leading causes of morbidity and mortality in infants and young children worldwide [3]. Respiratory syncytial virus (RSV) was the most frequently isolated virus (50–80%) and showed a consistent seasonal distribution in Greece from November to April, with peak incidence noted between January and March [3,4,5,6].

During the period of restrictive measures (2020–2021), a significant reduction in the incidence of viral bronchiolitis and other respiratory infections was reported globally, compared to the seasonal epidemic peaks in previous years, resulting in a decrease (40–99%) in emergency department (ED) visits and hospital admissions [7,8,9,10,11]. A key question is whether this decline in bronchiolitis cases during the pandemic primarily reflected the effect of restrictive measures, or whether some infants with subacute COVID-19 presentations were not classified as bronchiolitis. However, because systematic SARS-CoV-2 testing was implemented for all hospitalized children after March 2020 and detection rates were very low in our cohort, misclassification is unlikely to have substantially influenced the observed trends. By late 2020, concerns arose regarding the disruption of seasonal viral transmission patterns and the potential increase in the epidemic outbreaks risk during the upcoming seasons, once restrictive measures were lifted [12,13]. Epidemic models predicted that RSV outbreaks may occur during the winter seasons of 2021–2023 if non-pharmaceutical interventions remained in place for an extended period [13]. Indeed, evidence from several countries showed an increase in the bronchiolitis incidence during 2021–2022 compared to the pre-pandemic seasons, with outbreaks peaking earlier in the fall [14,15,16,17,18,19,20]. Data about the potential shifts in the severity of bronchiolitis during the pandemic remain controversial [15,19,21,22]. In Greece, data on the impact of the COVID-19 pandemic on the epidemiology of bronchiolitis are limited. To date, this is the first report to evaluate the impact of the COVID-19 pandemic on bronchiolitis epidemiology in Greece. This study aims to evaluate the effect of the COVID-19 pandemic on the epidemiology and disease severity among infants hospitalized with bronchiolitis over a 7-year.

## 2. Materials and Methods

A retrospective descriptive, non-interventional study was conducted at “P. and A. Kyriakou” Children’s Hospital in Athens, Greece, which is one of the two major tertiary care pediatric hospitals in Athens, serving as a referral hospital for the wider area of Athens as well as for the central and southern Greece.

### 2.1. Patient Selection

The electronic registry and medical records of all patients aged 0 to 24 months, who were hospitalized with a final diagnosis of bronchiolitis between 1 November 2017, and 30 September 2024, were reviewed. The clinical diagnosis of bronchiolitis was defined based on the National Institute for Health and Care Excellence (NICE) guidelines for infants <24 months [23].

### 2.2. Exclusion Criteria

Children who had experienced more than one previous episode of bronchiolitis, those with recurrent wheezing episodes due to viral infections, or children with a known history of asthma diagnosis were excluded from the study. Data on previous wheezing episodes were retrieved from electronic hospital records (including past admissions and outpatient visits) and systematically confirmed with parental history taken at admission.

Patients were also excluded in cases of a mismatch in recorded information or incomplete data. Finally, patients who were discharged before completing their hospitalization, as well as children with prolonged hospitalization due to another cause, such as possible nosocomial transmission, were excluded. The proportion of excluded patients was relatively small and comparable across seasons (8.9–11.1%). The study was approved by the institution’s Ethical and Research Committee (approval number: 507/20.10.2020). All procedures were conducted in accordance with the 1964 Declaration of Helsinki and its later amendments.

### 2.3. Data Collection

Data were collected from the patients’ medical and nursing records. An Excel database was used for data collection, entry and analysis, including demographic information and clinical characteristics, as well as data about the hospitalization, treatment, and outcome of the study population.

### 2.4. Virus Identification

Viral identification was performed at the time of hospital admission as part of routine clinical management, and results were retrieved from the patients’ electronic medical records. From 2017 to early 2020, testing primarily relied on rapid antigen detection using immunochromatographic assays (RSV, influenza A/B) on nasopharyngeal swabs and/or direct immunofluorescence using monoclonal antibodies in nasal washings detecting seven respiratory viruses [Influenza A & B, Respiratory Syncytial Virus, Adenovirus, Parainfluenza Virus—type 1, 2 & 3 (Respiratory Viral screening & identification Mab, ACRESI)]. For negative cases, molecular methods, such as Real-time Polymerase Chain Reaction—RT-PCR or Multiplex PCR/FilmArray [The BioFire^®^ Respiratory 2.1 (RP2.1) Panel, BioFire Diagnostics, bioMérieux, Marcy l (Etoile, France)] were performed, targeting a broad range of respiratory viruses and atypical bacteria. Since winter 2022–2023, a new molecular test in nasopharyngeal swabs, detecting RSV and/or Influenza A/B and/or SARS-CoV-2, has been used [Pluslife Influenza A/Influenza B/RSV/SARS-CoV-2 Nucleic Acid Test—Molecular POC analyzer Mini Dock PM001, Pluslife Biotech Co., Ltd., Guangzhou, China]. After March 2020, all hospitalized children were also systematically tested for SARS-CoV-2 using either rapid antigen test or RT-PCR in line with national infection-control protocols. Primer/probe sets targeted the envelope (E), nucleocapsid (N), and RNA-dependent RNA polymerase (RdRp) genes.

### 2.5. Assessment of Disease Severity

Disease severity was assessed using the following parameters: length of hospital stay, need for supplemental oxygen, type of respiratory support (low flow nasal oxygen—LFNO, high flow nasal cannula—HFNC, non-invasive support with continuous positive airway pressure—CPAP, mechanical ventilation—MV), duration of oxygen therapy, intensive care unit (ICU) admission, and length of ICU hospitalization. The Modified Tal Score (MTS), a validated clinical score for assessing the severity of bronchiolitis, was used [24]. Additionally, the presence of pulmonary infiltrates on chest X-ray, and readmission within one week after discharge were assessed.

### 2.6. Data Analysis by Time Periods

For data analysis, seven RSV seasons were examined from 1 November 2017, until 30 September 2024. The pre-pandemic period was defined as all admissions until 29 February 2020. Although SARS-CoV-2 was circulating globally before this date, the first confirmed cases in Greece and the introduction of restrictive measures occurred in early March 2020. Therefore, 1 March 2020 was used as the cut-off to distinguish the pre-pandemic from the pandemic period in our analysis. Therefore, the period between 1 November 2017, and 29 February 2020, represents the pre-COVID-19 period, and the period from 1 March 2020, until 30 April 2021, corresponds to the implementation of restrictive measures to address the COVID-19 pandemic. Finally, the period from 1 May 2021 to 30 September 2024, pertains to the “post-COVID-19 era” after the release of restrictive measures.

### 2.7. Statistical Analysis

Basic descriptive statistics were used to analyze data across seven time-reference groups: 2017–2018, 2018–2019, 2019–2020, 2020–2021, 2021–2022, 2022–2023, and 2023–2024. All data pertaining to the outcomes of the study were examined for normality using the Shapiro–Wilk’s test. Hence, categorical variables were expressed as absolute numbers (n) and percentages, while continuous and ordinal variables were presented as median and interquartile range (IQR)in cases of non-normal distribution or as mean and standard deviation in cases of normally distributed data.

Fischer’s exact test and chi-squared test were employed to investigate differences in the primary and secondary categorical outcomes of interest. Intergroup comparisons for continuous or ordinal variables were performed using the non-parametric Kruskal–Wallis test. Upon identifying a significant overall difference (*p* < 0.05), post hoc analyses were conducted to determine specific group differences. For continuous and ordinal variables, pairwise comparisons were performed using Dunn’s test. For categorical variables, pairwise comparisons were performed using Chi-square or Fisher’s exact tests. A Bonferroni correction was applied to all post hoc *p*-values to control the error rate induced by multiple comparisons. All statistical analyses were conducted with the SPSS software (IBM Corp. Released 2021. IBM SPSS Statistics for Windows, Version 28.0. Armonk, NY, USA: IBM Corp). A *p*-value less than 0.05 was considered statistically significant.

## 3. Results

### 3.1. Study Population

A total of 1505 patients hospitalized for bronchiolitis were included in the study. The median age at admission was 2.8 months (range 0.2–23.7), and 876 patients (58.2%) were males. Demographic characteristics and clinical presentation of the patients at admission are summarized in Table 1.

### 3.2. Viral Etiological Factors of Bronchiolitis Cases

Diagnostic testing, either antigenic or molecular, was performed in 98.1% of the patients. The distribution of pathogens across time periods is presented in Supplementary Table S1 (Online Resource). RSV was the predominant identified virus, detected in 62.2% of the patients. Since the beginning of the pandemic, SARS-CoV-2 was detected in 8 out of 1019 (0.8%) hospitalized children with bronchiolitis. Finally, a statistically significant rise in parainfluenza 3 (3.4%, *p* = 0.005), rhinovirus/enterovirus (10.9%, *p* < 0.001), adenovirus (4.1%, *p* < 0.001), and human-metapneumovirus (4.1%, *p* < 0.001) cases was observed during the 2023–2024 season.

**Figure 1:**
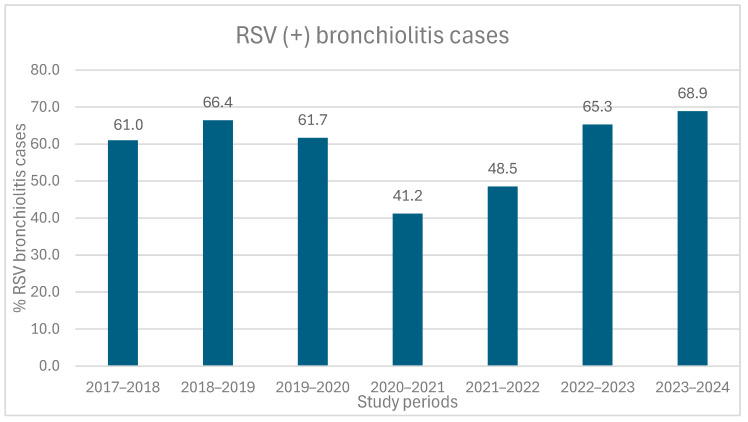
Proportion of RSV positive cases (expressed as percentages) among all bronchiolitis hospitalizations across the seven study periods (*p* < 0.001). Note: RSV detection decreased during the pandemic (2020–2021), partially rebounded in 2021–2022, and reached the highest levels in 2023–2024, similar to or above pre-pandemic seasons.

Figure 1 illustrates specifically the proportion of RSV-positive cases among all bronchiolitis hospitalizations across the seven study periods, showing a statistically significant difference between RSV seasons (*p* < 0.001). During the pandemic period of 2020–2021, only 28 cases (41.2%) of RSV positive bronchiolitis were recorded. With the gradual easing of restrictive measures from 2021 to 2022, 79 RSV positive bronchiolitis cases (48.5%) were recorded, showing a partial rebound. Ιn the 2022–2023 season, RSV was identified in 198 out of 303 hospitalized children (65.3%), comparable to pre-pandemic levels (61.0–66.4%). The highest rate of RSV positive bronchiolitis cases was recorded during the 2023–2024 season (68.9%).

Coinfections, defined as detection of more than one virus, were documented in 58 cases (3.8%). The majority involved combinations of RSV with rhinovirus/enterovirus, adenovirus, influenza or parainfluenza, while SARS-CoV-2 was detected in 8 children, 6 of whom had concomitant infection with another respiratory virus. Due to the small numbers and incomplete systematic multiplex testing in the earlier years, subgroup analyses of severity outcomes by coinfection status were not performed.

### 3.3. Seasonal Distribution

**Figure 2:**
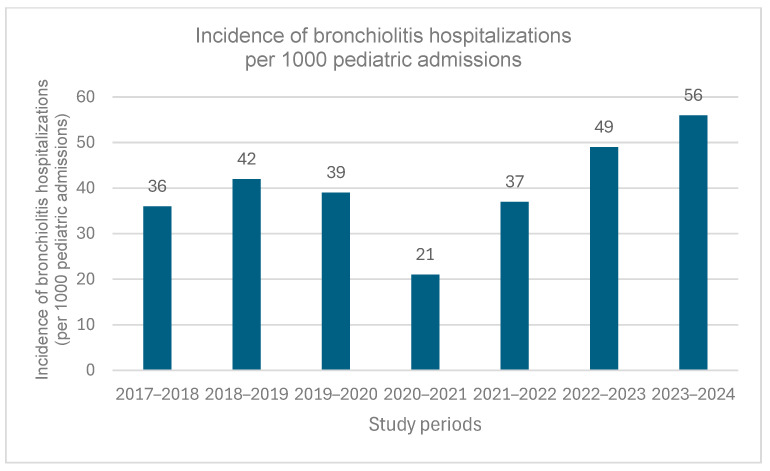
Incidence of bronchiolitis hospitalizations per 1000 pediatric admissions by study period (*p* < 0.001). Note: The incidence of bronchiolitis hospitalizations decreased markedly during the pandemic period (2020–2021), returned to pre-pandemic levels in 2022–2023, and exceeded pre-pandemic levels in 2023–2024.

Regarding the total number of hospitalizations for bronchiolitis, as shown in Table 1, the difference between seasonal periods was statistically significant (*p* < 0.001), with the lowest number observed during the 2020–2021 season (n = 74) and the highest during the 2022–2023 season (n = 305). However, the number of hospitalizations in 2022–2023 (n = 305) was comparable to that observed during the 2018–2019 season (n = 267), before the COVID-19 pandemic. Figure 2 shows the incidence of bronchiolitis hospitalizations per 1000 pediatric admissions by study period. A statistically significant variation was observed (*p* < 0.001), with the lowest incidence during 2020–2021 (21 per 1000 admissions) and the highest during 2023–2024 (56 per 1000 admissions). Incidence rates in 2022–2023 (49 per 1000 admissions) were comparable to those observed before the COVID-19 pandemic (36–42 per 1000 admissions during 2017 and 2020).

**Figure 3:**
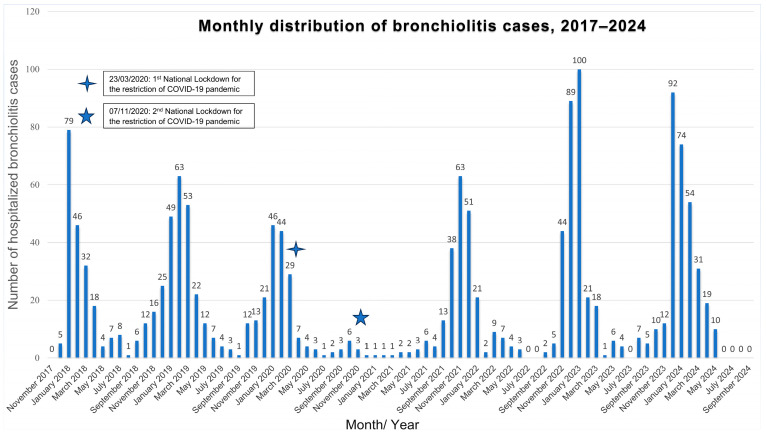
Monthly distribution of bronchiolitis cases during the study period (2017–2024). Note: Typical seasonal peaks occurred in January–February pre-pandemic, hospitalizations dropped sharply in 2020–2021, rebounded with an early peak in 2021–2022, and returned to pre-pandemic patterns in 2022–2024, with the highest peak observed in January 2023. All differences observed in the monthly hospitalizations due to bronchiolitis between the different study periods were statistically significant (*p* < 0.001), except for May and June, where no statistically significant differences were reported (*p* = 0.74 and *p* = 0.73, respectively). “Four-pointed star” indicates the date of 1^st^ national lockdown for the restriction of COVID-19 pandemic; “Five-pointed star” indicates the date of 2^nd^ national lockdown for the restriction of COVID-19 pandemic.

The overall seasonal distribution of new bronchiolitis cases hospitalized from 1 November 2017 to 30 September 2024 is shown in Figure 3.

During the 2017–2020 seasons, the expected seasonal distribution of bronchiolitis cases was observed; it typically began in October, peaked during January and February, and declined by April–May. At the pandemic’s onset in March 2020, hospitalizations decreased sharply following the implementation of restrictive measures (23 March 2020). Only 12 bronchiolitis admissions were recorded in fall 2020, primarily in October (n = 6; 1.8%). After the second lockdown (7 November 2020), admissions remained low, with no seasonal peak. Bronchiolitis hospitalizations declined by nearly 98% compared to previous RSV seasons.

Unlike previous seasons, in 2021–2022, an early surge in bronchiolitis cases was observed, peaking in November (n = 63; 19.2% of admissions that month) and December (n = 51; 15.8%), before abruptly declining from January to February (n = 21 and 2, respectively). During the summer of 2021, 13 inter-seasonal hospitalizations occurred between June and August, following the gradual easing of restrictions.

In the 2022–2023 and 2023–2024 seasons, the seasonal distribution of bronchiolitis cases returned to the pre-pandemic patterns. The first cases appeared in late October (n = 5–10; 1.0–2.1%), increased significantly in November and December (n = 44–92; 7.1–15.2%), and peaked in January 2023 (n = 100; 16.0%) and in December 2023 (n = 92; 15.2%). Afterward, cases gradually declined until late spring (April–May: n = 1–19; 0.8–3.6%). The highest hospitalization peak was observed in January 2023 (100 cases, 16% of pediatric admissions), surpassing the 2017–2020 pre-pandemic average by 55.5% (ranging from 24 to 113% above each corresponding season).

All differences observed in the monthly hospitalizations due to bronchiolitis between the different study periods were statistically significant (*p* < 0.001), except for May and June, where no statistically significant differences were reported (*p* = 0.74 and *p* = 0.73, respectively) (see Supplementary Table S2, Online Resource).

### 3.4. Age Distribution

**Figure 4:**
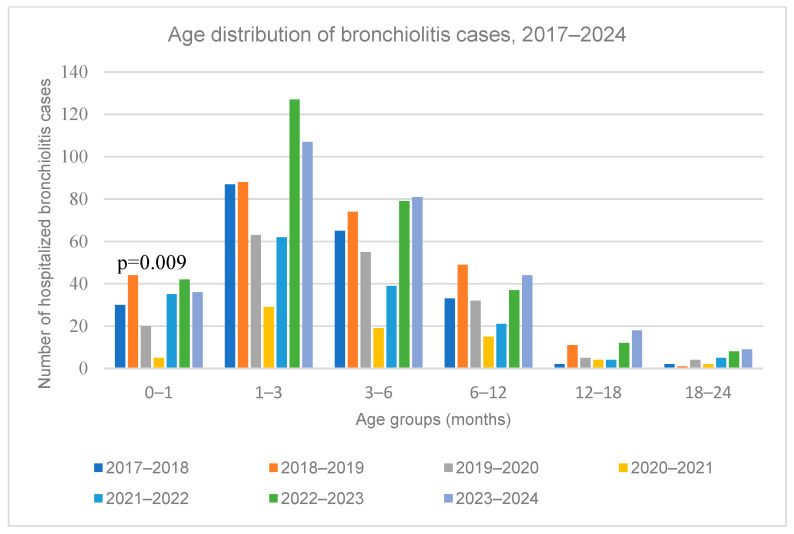
Age group distribution of bronchiolitis hospitalizations across study periods (2017–2024). Note: Infants aged 1–3 months consistently had the highest hospitalization rates. The lowest number of newborn admissions occurred during the 2020–2021 pandemic period.

The median age of hospitalized bronchiolitis cases did not differ significantly across the periods (*p* = 0.18) (Table 1). The age distribution by period is shown in Figure 4. Infants aged 1–3 months had the highest hospitalization rates (33.0–41.6%). A statistically significant difference was observed in 2020–2021, when the lowest number of hospitalizations in newborns with bronchiolitis was recorded (5 newborns, 6.8% (*p* = 0.009) (see Supplementary Table S2, Online Resource), reflecting the overall decline in bronchiolitis admissions during the period of strict pandemic restrictions. This reduction appeared uniform, with no evidence that any specific age group was disproportionately affected.

### 3.5. Disease Severity

Disease severity, assessed by the Modified Tal Score and other indirect factors, is reported in Table 2. The median hospital stay was 4 days. Overall, 1251 out of 1505 hospitalized children (83.1%) required respiratory support for a median duration of 4 days (range 1–25), with only 4 patients (0.4%) requiring intubation, all before the emergence of the pandemic. A statistically significant less frequent need for respiratory support was observed during 2020–2021 and 2021–2022 (78.4% and 75.8%, respectively, *p* < 0.001), while in 2022–2023 and 2023–2024, the proportion of bronchiolitis cases who were administered supplementary oxygen (81.6% and 83.1%, respectively) was similar to the pre-pandemic levels (81.6–88.9%). No difference was noted in the duration of oxygen therapy. HFNC use increased in 2023–2024 compared to other periods (15.9% vs. 3.2–8.6%, *p* < 0.001), with a median longer duration in 2022–2024 (4 days vs. 2–3 days, *p* < 0.001). Nevertheless, no statistically significant differences were found in hospital stay, ICU admissions, length of ICU stay, the rate and duration of CPAP/mechanical ventilation support, and readmissions across the periods. No deaths occurred in the study population. During the 2017–2024 period, the median MTS at admission was 5 (range 1–12), with a peak score of 6 (range 1–12). Correlation analysis between MTS (at admission and peak) and time period did not reveal statistical significance (*p* = 0.52 and *p* = 0.41, respectively). In the post-pandemic period (2021–2024), pulmonary infiltrates on chest X-ray increased significantly (49.2%, 32.4%, and 38.7%) compared to 2017–2021 (*p* < 0.001) (Table 2).

When restricting the analysis to more objective indicators of disease severity, no significant differences were observed across study years. ICU admission rates ranged from 1.4% to 8.1% (overall 3.8%), without a consistent temporal trend. Intubation was required in only four cases (0.3%), all occurring before the COVID-19 pandemic, with none recorded during or after 2020. These findings suggest that, despite changes in management practices such as the introduction of HFNC, there was no evidence of increased severity when assessed by objective endpoints.

Finally, a subgroup analysis restricted to infants ≤12 months (Supplementary Table S4) yielded results nearly identical to the primary analysis, confirming these findings.

## 4. Discussion

This study reports changes in bronchiolitis hospitalizations among Greek children following the COVID-19 pandemic. During the pandemic, restrictive public health measures were associated with a reduction in bronchiolitis cases globally [12]. With the COVID-19 vaccines availability and the gradual release of restrictions, a resurgence of bronchiolitis and RSV infections was expected [13]. This shift may be attributed to the “immunological debt” caused by reduced exposure to common viruses, leading to a lack of immune memory and increased susceptibility to infections [12,14,15,16,17,18,19,20]. Also, other factors, including delayed care-seeking, changes in healthcare-seeking behavior, evolving diagnostic technologies, and viral–viral interactions, must also be considered when interpreting these findings.

During the 2020–2021 pandemic restrictions, bronchiolitis hospitalizations in our settings declined by up to 98% across all age groups compared to previous years, supporting the overall suppression of viral transmission during lockdown measures, rather than an age-specific effect. With the gradual lifting of restrictions, bronchiolitis hospitalizations returned to pre-pandemic levels. However, the proportion of bronchiolitis cases among total pediatric admissions increased from 4.9% in 2022–2023 to 5.6% in 2023–2024, indicating a notable increase compared to the pre-pandemic levels (3.9% for the periods 2017–2020). This trend suggests a heightened relative impact of bronchiolitis on pediatric admissions in the post-pandemic period. The findings of this study are in line with the global observations [14,16,17,20,25], while other studies report incidence rates higher than pre-pandemic levels [15,18]. A shift in seasonal distribution was also observed, with cases starting earlier in autumn and peaking in November, contrary to the typical patterns. In 2022–2023, bronchiolitis hospitalizations peaked, increasing by 58.7% compared to pre-pandemic peaks. By 2023–2024, the seasonal distribution gradually returned to pre-pandemic patterns. Evidence from Italy indicated a higher incidence in 2021–2022 [15,26], followed by a return to pre-pandemic patterns in 2022–2023 [26]. This difference aligns with predictive models [13], which showed that prolonged nonpharmaceutical interventions could delay and amplify RSV resurgence by increasing the pool of susceptible individuals. Specifically, in Italy, where restrictions were lifted earlier, the peak occurred in 2021–2022, whereas in Greece, where measures remained in place for a longer period, the peak was delayed until 2022–2023.

In terms of the age distribution of bronchiolitis cases, no change was observed during or after the pandemic, with infants aged 1–3 months accounting for the majority of hospitalizations. However, while only 5 neonates were hospitalized during the first pandemic year, hospitalizations among neonates and infants aged 1–3 months increased after the lifting of restrictions, a trend that was also reported in Italy [26]. A plausible assumption is that reduced RSV exposure led to lower maternal immunity and therefore, reduced neonatal protection [27]. The lower neonatal incidence during the pandemic may be due to the protective effect of pre-existing maternal and household immunity, providing a “cocooning”-like protection [28,29], combined with reduced exposure of infants to social contacts due to the parental fear of the pandemic [30].

An interesting finding was the temporary decline in the proportion of premature infants hospitalized with bronchiolitis between 2019 and 2022, followed by an increase in subsequent years. This likely reflects the indirect effects of pandemic restrictions, which reduced viral circulation and exposure in this particularly vulnerable group. Similar reductions in RSV hospitalizations among preterm infants during the pandemic period have been previously reported [31]. In addition, parental hesitancy to seek care during the pandemic may have further contributed to the decline of bronchiolitis admissions among premature infants [32]. The rebound after 2022 is consistent with the lifting of restrictions and the return of viral circulation to pre-pandemic levels, highlighting how social measures can influence high-risk populations [33].

From a microbiological perspective, RSV was the most common virus throughout the study period. RSV activity remained low at the beginning of the pandemic (41.2%), but increased in 2022–2023 and 2023–2024 (65.3% and 68.9%, respectively), reaching the pre-pandemic levels, consistent with previous studies [34]. Despite systematic SARS-CoV-2 screening after March 2020, the virus was detected in only 8 out of 1019 children hospitalized for bronchiolitis (0.8%). In 6 of these cases, a co-infection with other respiratory viruses was observed. None of these children required ICU admission or HFNC, confirming that SARS-CoV-2 is not an important cause of bronchiolitis and is not linked to severe disease [15,16,21,25,35,36,37]. This observation is consistent with international multicenter studies, which similarly report that bronchiolitis caused by SARS-CoV-2 in infants is uncommon, generally mild, and not associated with increased severity compared to RSV or other viral pathogens [35,36,38]. Therefore, these findings support the conclusion that SARS-CoV-2 is unlikely to act as a major bronchiolitis pathogen in young children. Other viruses, including rhinovirus/enterovirus, adenovirus, parainfluenza 3, and human-metapneumovirus, gained prominence in 2023–2024, possibly due to viral competition and the effect of reduced restrictions [39]. However, it is worth noting that the broader use of advanced pathogen detection methods, such as FilmArray, in recent years may have contributed to the increased detection of these viruses.

Regarding disease severity, during the first pandemic year, the need for respiratory support declined, possibly linked to reduced RSV circulation. In 2023–2024, a notable increase in HFNC use and duration was observed, likely influenced by a change in medical practices, as HFNC became available on the ward in December 2022, whereas previously, children requiring HFNC were admitted to the ICU. However, no significant changes in the incidence of ICU admissions, mechanical ventilation, hospital stay length, and readmission rates were observed, which aligns with a study conducted in Italy, suggesting that this change reflects a more aggressive treatment approach after the pandemic rather than an increase in disease severity [21]. No significant difference in MTS was noted across periods, either at admission or during the peak disease.

A notable increase in pulmonary infiltrates on chest x-rays was observed after the restrictions lifting, especially in 2021–2022, supporting the “immunity debt” theory [12,40]. During the period of restrictive measures, a significant reduction in pneumonia and invasive streptococcal infections was reported, likely due to decreased bacterial circulation [12]. However, recent studies disproved a decrease in pneumococcal carriage and showed a decline in invasiveness due to the absence of viral infections, reduced exposure and delayed vaccinations, which may have led to increased secondary bacterial infections in 2021–2023 [40,41]. Moreover, the increase in pulmonary infiltrates in 2023–2024 may be attributed to the increased circulation of non-RSV pathogens, such as human-metapneumovirus and parainfluenza 3, which are associated with a more severe disease course than RSV [42]. Overall, post-COVID-19 bronchiolitis severity is equivocal, with most studies reporting higher incidence without a significant change in severity [15,16,17,18,25,26,43,44,45], while other studies demonstrate more severe cases, particularly in 2022–2023 [19,46,47,48,49]. These variations may reflect regional climate differences, variations in population susceptibility, and changes in social behaviors during the pandemic [13].

In addition, our results highlight the importance of prevention. The recent introduction of maternal RSV vaccination during pregnancy and the availability of long-acting monoclonal antibodies, such as nirsevimab, result in reducing RSV-related morbidity and hospitalizations, particularly in the first months of life when disease burden is increased [50,51,52]. Both clinical trial and early real-world evidence demonstrate that these interventions substantially reduce RSV hospitalizations [50,51,52]. Their incorporation into national immunization programs, combined with virological surveillance, will be essential for optimizing outcomes and informing healthcare policy about the impact of these strategies.

At the same time, the COVID-19 pandemic highlighted the influence of social and non-pharmaceutical measures on viral circulation, demonstrating how human behavior can reshape established epidemiological patterns [33]. Beyond traditional surveillance, modern systems biology approaches, such as weighted gene co-expression network analysis of bronchial epithelial transcriptional profiles, combined with explainable artificial intelligence, have been used to identify host–pathogen interactions and novel therapeutic targets [53]. Integrating such molecular and computational insights with population-level epidemiology will be critical in the post-pandemic era, resulting in the development of preventive strategies, such as maternal RSV vaccination and long-acting monoclonal antibodies, and more targeted therapeutic interventions for viral respiratory infections.

The present study has certain limitations. First, this is a retrospective study. However, an extensive search of hospital electronic records suggests that almost all patients admitted for bronchiolitis were identified. A small proportion of medical records could not be retrieved (missing data: 8.8–9.8% across epidemic seasons), and excluded cases due to predefined clinical criteria represented 8.9–11.1% per season. Both missing and excluded proportions were relatively low and consistently distributed across study periods, minimizing the likelihood of introducing systematic selection bias. Moreover, data collection relied on multiple health professionals’ records, which may pose a risk of information bias. Nonetheless, the use of multiple consistent entries enhances reliability, and careful exclusion of mismatched data ensures the integrity of the analyzed dataset. In addition, another limitation is the single-center design. Although our hospital is one of the two major tertiary pediatric referral centers in Athens and covers a large area that includes central and southern Greece, its patient population may not fully represent the entire national pediatric population. Regional variations in demographics, adherence to restrictive measures and healthcare access, could have influenced bronchiolitis epidemiology. Nevertheless, our findings align with recently published Greek surveillance data on RSV circulation, which supports the representativeness of our observations at a national level [34].

Patient selection is another limitation. This study set the upper age cut-off for bronchiolitis at 24 months according to NICE criteria, as there is no international consensus, though some authors use 12 months as the upper cutoff. [22,54]. This might raise the concern that older infants with early wheezing disorders were falsely categorized as bronchiolitis, overestimating its incidence. However, cases with more than one previous episode of bronchiolitis were excluded to avoid mistakenly including any cases of recurrent wheezing or early asthma. Therefore, with the use of the clinical definition of bronchiolitis, this limitation was overcome. Also, we performed a subgroup analysis restricted to infants ≤12 months (Supplementary Table S4), which represented 94% of the original cohort. The results were nearly identical to those of the main analysis, supporting the robustness of our conclusions. A further limitation is the evolution of diagnostic methods over time. During the early years, antigen detection and immunofluorescence were the main diagnostic tools, while multiplex PCR and point-of-care assays were introduced during later years, which may partly explain the apparent rise in non-RSV pathogens. However, RSV was consistently tested across all seasons and thus, provides the most reliable basis for longitudinal comparison, while non-RSV findings should be interpreted with caution.

Additionally, while univariate analyses revealed temporal trends in disease severity, we cannot rule out residual confounding from factors such as prematurity or comorbidities; future studies with a dedicated risk-factor design are warranted to isolate the independent effect of the pandemic era. Finally, assessment of disease severity relied mainly on indirect indicators such as length of stay, oxygen requirement, and the Modified Tal Score, which may not fully capture subtle changes. Moreover, changes in clinical practices, particularly the introduction of HFNC therapy in 2022–2023, likely influenced management and may limit comparability across years. To address this, we performed a subanalysis of more objective outcomes, such as ICU admissions, intubation and mortality, which remained stable across periods and therefore provide a more reliable basis for longitudinal comparison.

## 5. Conclusions

The COVID-19 pandemic has significantly altered the epidemiology of bronchiolitis in Greece, increasing the strain to the healthcare system. These epidemiological changes in bronchiolitis following the COVID-19 pandemic should be interpreted as the outcome of multiple factors. These factors range from population-level immunity gaps and viral competition to evolving diagnostic practices and healthcare behaviors, rather than the effect of a single determinant.

Close monitoring and surveillance of RSV, SARS-CoV-2, and other respiratory viruses is essential for optimizing resource allocation and healthcare policies. With the recent introduction of maternal RSV vaccination and new monoclonal antibodies, such as nirsevimab, monitoring their impact on RSV circulation will be crucial for developing effective prevention strategies and reducing the disease burden of bronchiolitis.

## Figures and Tables

**Table 1:** Demographic and clinical characteristics of patients hospitalized with acute bronchiolitis during the 2017–2024 study period.

	RSV Seasons							
RSV seasons	2017–2018	2018–2019	2019–2020	2020–2021	2021–2022	2022–2023	2023–2024	2017–2024
Patients								
Total hospitalized bronchiolitis patients, (per 1000 pediatric admissions)	219 (36)	267 (42)	179 (39)	74 (21)	166 (37)	305 (49)	295 (56)	1505 (41) ^d^
Total admissions in PED departments	6047	6300	4572	3518	4531	6236	5222	36,426
Age (months)	2.8 (3.2)	3 (4.2)	3.1 (4.2)	3 (6.0)	2.63 (3.2)	2.53 (3.9)	3.13 (4.3)	2.80 (3.7)
Gender (M/F)	139/80	160/107	104/75	52/22	84/82	161/144	178/117	876/627
Parity	2 (2)	2 (1)	2 (1)	2 (2)	2 (1)	2 (1)	2 (2)	2 (1)
Prematurity n, (%)	47 (23.5)	53 (20.5)	20 (11.6)	19 (26.4)	36 (22.2)	66 (21.6)	54 (18.3)	295/1505 (19.6)
Previous hospitalization for bronchiolitis n, (%)	27 (12.9)	37 (14)	25 (14.5)	9 (12.5)	12(7.3)	35 (11.5)	40 (13.6)	185/1505 (12.3)
Comorbidities n, (%) ^a^	14 (6.4)	23 (8.6)	12 (6.7)	2 (2.7)	8 (4.8)	12 (3.9)	18 (6.1)	89/1505 (5.9)
Clinical presentation								
Respiratory distress ^b^	161 (74.2)	199 (74.8)	141 (79.2)	66 (88.0)	145 (87.3)	259 (84.9)	230 (78)	1201 (79.8)
Respiratory Rate on admission	53 (15)	56 (17)	56 (15)	52 (15)	56 (15)	56 (10)	52 (15)	55 (15)
SpO_2_ (%) on admission	97 (4)	97 (4)	96.5 (5)	98 (5)	97 (5)	96 (4)	97 (4)	97 (5)
Heart Rate on admission	150 (30)	150 (24)	150 (28)	150 (22)	154 (30)	152 (29)	154 (28)	150 (29)
Fever >38 °C ^c^ (°C)	82 (39.4)	140 (53.2)	98 (55.1)	39 (52.7)	73 (44.2)	123 (40.3)	95 (31.1)	650 (43.2)
Maximum temperature ^c^ (°C)	38.4 (1.1)	38.4 (1.1)	38.4 (1.2)	38.6 (0.9)	38.5 (1.1)	38.5 (1.3)	38.5 (1.1)	38.5 (1.1)
Apnoea episodes ^c^	22 (10.2)	14 (5.3)	4 (2.3)	0	9 (5.4)	13 (4.3)	5 (1.7)	67 (4.5)

Values are presented as median (IQR). The percentages have been calculated by reduction to the total available per variable data. ^a^ Comorbidities are defined as the presence of one or more of the following: bronchopulmonary dysplasia or other chronic lung disease, congenital heart disease, genetic disorder (Trisomy 21 and others), neuromuscular disorder, haematologic and/or oncologic disease, and immunodeficiency disorder. ^b^ Respiratory distress is defined as the presence of tachypnea and/or retractions during clinical examination. ^c^ Either prior to hospitalization (as reported in the medical history) or recorded during the hospital stay. ^d^ Statistically significant difference among the seasonal periods, regarding the proportion of bronchiolitis cases out of the total admissions in the pediatric departments of the hospital (*p* < 0.001). Abbreviations: M/F, male/female; SpO_2_, peripheral oxygen saturation.

**Table 2:** Disease severity of bronchiolitis cases, assessed by Modified Tal Score (MTS), respiratory support requirements, and outcomes among patients hospitalized for acute bronchiolitis during the 2017–2024 study period.

	2017–2018	2018–2019	2019–2020	2020–2021	2021–2022	2022–2023	2023–2024	2017–2024	*p*-Value
Total	219	267	179	74	166	305	295	1505	
Admission MTS	5 (4)	5 (3)	5 (3)	5 (4)	5 (2)	5 (2)	5 (3)	5 (2)	0.52
Peak MTS	6 (3)	6 (2)	6 (3)	5 (3)	5 (3)	6 (2)	6 (3)	6 (3)	0.41
Requirement for respiratory support n, (%)	193 (88.9)	228 (85.4)	146 (81.6)	58 (78.4)	125 (75.8)	256 (81.6)	245 (83.1)	1251 (83.1)	<0.001
Type of respiratory support ^a^ n, (%)									
LFNO	193 (100)	228 (100)	146 (100)	58 (100)	125 (100)	256 (100)	241 (98.4)	1247 (99.7)	0.7
HFNC	12 (6.2)	8 (3.5)	7 (4.8)	5 (8.6)	4 (3.2)	15 (5.6)	39 (15.9)	90 (7.2)	<0.001
CPAP	5 (2.6)	1 (0.4)	1 (0.7)	0	0	2 (0.8)	0	9 (0.7)	0.38
MV	1 (0.5)	1 (0.4)	2 (1.4)	0	0	0	0	4 (0.3)	0.82
Duration of respiratory support (days)	4 (4)	4 (3)	4 (3)	3 (4)	4 (4)	3 (3)	3 (3)	4 (3)	0.25
Duration of HFNC use (days)	3 (0)	2 (0)	2 (0)	2,5(0)	3 (0)	4 (0)	4 (4)	3 (1)	<0.001
Duration of hydration (days)	3 (4)	2 (3.7)	2 (3)	2 (3)	2 (4)	2 (3)	3 (3)	2 (3)	0.31
ICU admission n, (%)	16 (7.5)	9 (3.4)	7 (3.9)	6 (8.1)	6 (3.6)	9 (2.9)	4 (1.4)	57 (3.8)	0.03
Length of stay in ICU (days)	4 (0)	3 (0)	4 (0)	2.5(0)	3 (0)	4 (0)	7 (0)	3 (0)	0.16
Length of Hospital Stay (days)	5 (4)	4 (3)	4 (3)	5 (5)	4 (3)	4 (3)	4 (3)	4 (4)	0.38
Hospital Readmission ^b^ n, (%)	1 (0.4)	7 (2.6)	4 (2.2)	1 (1.3)	1 (0.6)	7 (2.3)	3 (1)	20 (1.7)	0.68
Chest X-ray performed n, (%)	124 (56.6)	116 (43.4)	76 (42.5)	29 (39.2)	63 (37.9)	105 (34.4)	124 (42)	637 (42.3)	<0.001
Findings in Chest X-ray n, (%) ^c^	111 (89.5)	101 (87.1)	71 (93.4)	27 (89.6)	56 (88.9)	96 (91.4)	111 (89.5)	573 (90)	0.95
Pulmonary infiltrates n, (%) ^c^	19 (15.3)	24 (20.7)	20 (26.3)	7 (24.1)	31 (49.2)	34 (32.4)	48 (38.7)	183 (28.7)	<0.001

Values are presented as median (IQR). The percentages have been calculated by reduction to the total available per variable data. ^a^ Percentages are calculated over the total cases that required respiratory support. ^b^ Readmission within a week due to bronchiolitis following the initial discharge. ^c^ Percentages are calculated over the total cases where a chest X-ray was performed. Abbreviations: MTS, Modified Tal Score [24]; LFNO, Low Flow Nasal Oxygen; HFNC, High Flow Nasal Cannula; CPAP, Non-invasive support with continuous positive airway pressure; MV, Mechanical Ventilation; ICU, Intensive Care Unit.

## Data Availability

The data underlying this article will be shared on reasonable request to the corresponding author.

## Supplementary Materials

The following supporting information can be downloaded at: https://www.mdpi.com/article/10.3390/medicina61101746/s1, Table S1: Pathogenic agents detected in bronchiolitis cases, over the study periods 2017–2024.; Table S2: Monthly distribution of bronchiolitis cases during the study period, 2017–2024.; Table S3: Number of bronchiolitis cases per age group by study period from 2017 to 2024.; Table S4. Subgroup analyses for patients ≤12 months of age at presentation.

## Abbreviations

RSV	Respiratory Syncytial Virus
SARS-CoV-2	Severe Acute Respiratory Syndrome Coronavirus 2
ED	Emergency Department
RT-PCR	Reverse Transcription Polymerase Chain Reaction
LFNO	Low-Flow Nasal Oxygen
HFNC	High-Flow Nasal Cannula
CPAP	Continuous Positive Airway Pressure
MV	Mechanical Ventilation
ICU	Intensive Care Unit
MTS	Modified Tal Score
SpO_2_	Peripheral Capillary Oxygen Saturation
NICE	National Institute for Health and Care Excellence

## References

[1] WHO Coronavirus Disease (COVID-19) Pandemic_Timeline: WHO’s COVID-19 Response. n.d. https://www.who.int/emergencies/diseases/novel-coronavirus-2019/interactive-timeline.

[2] Greek National Public Health Organization Greek National Public Health Organization_Press Releases 2020–2023. n.d. https://eody.gov.gr/category/deltia-typoy/.

[3] Florin T.A., Plint A.C., Zorc J.J. (2017). Viral bronchiolitis. Lancet.

[4] Pogka V., Kossivakis A., Kalliaropoulos A., Moutousi A., Sgouras D., Panagiotopoulos T., Chrousos G.P., Theodoridou M., Syriopoulou V.P., Mentis A.F. (2011). Respiratory viruses involved in influenza-like illness in a Greek pediatric population during the winter period of the years 2005–2008. J. Med. Virol..

[5] Moriyama M., Hugentobler W.J., Iwasaki A. (2020). Seasonality of Respiratory Viral Infections. Annu. Rev. Virol..

[6] Tsolia M.N., Kafetzis D., Danelatou K., Astra H., Kallergi K., Spyridis P., Karpathios T.E. (2002). Epidemiology of respiratory syncytial virus bronchiolitis in hospitalized infants in Greece. Eur. J. Epidemiol..

[7] Van Brusselen D., De Troeyer K., ter Haar E., Vander Auwera A., Poschet K., Van Nuijs S., Bael A., Stobbelaar K., Verhulst S., Van Herendael B. (2021). Bronchiolitis in COVID-19 times: A nearly absent disease?. Eur. J. Pediatr..

[8] Markham J.L., Richardson T., DePorre A., Teufel R.J., Hersh A.L., Fleegler E.W., Antiel R.M., Williams D.C., Goldin A.B., Shah S.S. (2021). Inpatient Use and Outcomes at Children’s Hospitals During the Early COVID-19 Pandemic. Pediatrics.

[9] Chiapinotto S., Sarria E.E., Mocelin H.T., Lima J.A.B., Mattiello R., Fischer G.B. (2021). Impact of non-pharmacological initiatives for COVID-19 on hospital admissions due to pediatric acute respiratory illnesses. Paediatr. Respir. Rev..

[10] Stera G., Pierantoni L., Masetti R., Leardini D., Biagi C., Buonsenso D., Pession A., Lanari M. (2021). Impact of SARS-CoV-2 Pandemic on Bronchiolitis Hospitalizations: The Experience of an Italian Tertiary Center. Children.

[11] Guedj R., Lorrot M., Lecarpentier T., Leger P., Corvol H., Carbajal R. (2021). Infant bronchiolitis dramatically reduced during the second French COVID-19 outbreak. Acta Paediatr..

[12] Cohen R., Ashman M., Taha M.-K., Varon E., Angoulvant F., Levy C., Rybak A., Ouldali N., Guiso N., Grimprel E. (2021). Pediatric Infectious Disease Group (GPIP) position paper on the immune debt of the COVID-19 pandemic in childhood, how can we fill the immunity gap?. Infect. Dis. Now..

[13] Baker R.E., Park S.W., Yang W., Vecchi G.A., Metcalf C.J.E., Grenfell B.T. (2020). The impact of COVID-19 nonpharmaceutical interventions on the future dynamics of endemic infections. Proc. Natl. Acad. Sci. USA.

[14] Billard M.-N., Bont L.J. (2023). Quantifying the RSV immunity debt following COVID-19: A public health matter. Lancet Infect. Dis..

[15] Brisca G., Mariani M., Buratti S., Ferretti M., Pirlo D., Buffoni I., Mallamaci M., Salvati P., Tagliarini G., Piccotti E. (2023). How has the SARS-CoV-2 pandemic changed the epidemiology and management of acute bronchiolitis?. Pediatr. Pulmonol..

[16] Camporesi A., Morello R., Ferro V., Pierantoni L., Rocca A., Lanari M., Trobia G.L., Sciacca T., Bellinvia A.G., De Ferrari A. (2022). Epidemiology, Microbiology and Severity of Bronchiolitis in the First Post-Lockdown Cold Season in Three Different Geographical Areas in Italy: A Prospective, Observational Study. Children.

[17] Faraguna M.C., Lepri I., Clavenna A., Bonati M., Vimercati C., Sala D., Cattoni A., Melzi M.L., Biondi A. (2023). The bronchiolitis epidemic in 2021–2022 during the SARS-CoV-2 pandemic: Experience of a third level centre in Northern Italy. Ital. J. Pediatr..

[18] Vaux S., Viriot D., Forgeot C., Pontais I., Savitch Y., Barondeau-Leuret A., Smadja S., Valette M., Enouf V., Parent du Chatelet I. (2022). Bronchiolitis epidemics in France during the SARS-CoV-2 pandemic: The 2020–2021 and 2021–2022 seasons. Infect. Dis. Now..

[19] Mrcela D., Markic J., Zhao C., Viskovic D.V., Milic P., Copac R., Li Y. (2022). Changes following the Onset of the COVID-19 Pandemic in the Burden of Hospitalization for Respiratory Syncytial Virus Acute Lower Respiratory Infection in Children under Two Years: A Retrospective Study from Croatia. Viruses.

[20] Ujiie M., Tsuzuki S., Nakamoto T., Iwamoto N. (2021). Resurgence of Respiratory Syncytial Virus Infections during COVID-19 Pandemic, Tokyo, Japan. Emerg. Infect. Dis..

[21] Ghirardo S., Cozzi G., Tonin G., Risso F.M., Dotta L., Zago A., Lupia D., Cogo P., Ullmann N., Coretti A. (2022). Increased use of high-flow nasal cannulas after the pandemic in bronchiolitis: A more severe disease or a changed physician’s attitude?. Eur. J. Pediatr..

[22] Cardenas J., Pringle C., Filipp S.L., Gurka M.J., Ryan K.A., Avery K.L. (2022). Changes in Critical Bronchiolitis After COVID-19 Lockdown. Cureus.

[23] NICE (2015). NICE Guideline NG9_Bronchiolitis: Diagnosis and Management of Bronchiolitis in Children, Methods, Evidence and Recommendations, June 2015-Updated August 2021. https://www.nice.org.uk/Guidance/NG9.

[24] Golan-Tripto I., Goldbart A., Akel K., Dizitzer Y., Novack V., Tal A. (2018). Modified Tal Score: Validated score for prediction of bronchiolitis severity. Pediatr. Pulmonol..

[25] Jiang M., Xu Y., Wu H., Zhu R., Sun Y., Chen D.M., Wang F., Zhou Y.T., Guo Q., Wu A. (2023). Changes in endemic patterns of respiratory syncytial virus infection in pediatric patients under the pressure of nonpharmaceutical interventions for COVID-19 in Beijing, China. J. Med. Virol..

[26] Camporesi A., Morello R., Pierucci U.M., Proli F., Lazzareschi I., Bersani G., Valentini P., Roland D., Buonsenso D. (2023). 2021/22 and 2022/23 Post-Pandemic Bronchiolitis Seasons in Two Major Italian Cities: A Prospective Study. Children.

[27] Juhn Y.J., Wi C.-I., Takahashi P.Y., Ryu E., King K.S., Hickman J.A., Yao J.D., Binnicker M.J., Natoli T.L., Evans T.K. (2023). Incidence of Respiratory Syncytial Virus Infection in Older Adults Before and During the COVID-19 Pandemic. JAMA Netw. Open.

[28] Ogilvie M.M., Santhire Vathenen A., Radford M., Codd J., Key S. (1981). Maternal antibody and respiratory syncytial virus infection in infancy. J. Med. Virol..

[29] Graham B.S. (2014). Protecting the Family to Protect the Child: Vaccination Strategy Guided by RSV Transmission Dynamics. J. Infect. Dis..

[30] Ryan L., Plötz F.B., van den Hoogen A., Latour J.M., Degtyareva M., Keuning M., Klingenberg C., Reiss I.K.M., Giannoni E., Roehr C. (2022). Neonates and COVID-19: State of the art: Neonatal Sepsis series. Pediatr. Res..

[31] Principi N., Autore G., Ramundo G., Esposito S. (2023). Epidemiology of Respiratory Infections during the COVID-19 Pandemic. Viruses.

[32] Zee-Cheng J.E., McCluskey C.K., Klein M.J., Scanlon M.C., Rotta A.T., Shein S.L., Pineda J.A., Remy K.E., Carroll C.L. (2021). Changes in Pediatric ICU Utilization and Clinical Trends During the Coronavirus Pandemic. Chest.

[33] Agha R., Avner J.R. (2021). Delayed Seasonal RSV Surge Observed During the COVID-19 Pandemic. Pediatrics.

[34] Berikopoulou M.M., Dessypris N., Kalogera E., Petridou E., Benetou V., Zahariadou L.D., Siahanidou T., Michos A. (2024). Epidemiology of respiratory syncytial virus in hospitalized children before, during, and after the COVID-19 lockdown restriction measures in Greece. Epidemiol. Infect..

[35] Cozzi G., Sovtic A., Garelli D., Krivec U., Silvagni D., Corsini I., Colombo M., Giangreco M., Giannattasio A., Milani G.P. (2023). SARS-CoV-2-related bronchiolitis: A multicentre international study. Arch. Dis. Child..

[36] Andina-Martinez D., Alonso-Cadenas J.A., Cobos-Carrascosa E., Bodegas I., Oltra-Benavent M., Plazaola A., Epalza C., Jimenez-García R., Moraleda C., Tagarro A. (2022). SARS-CoV-2 acute bronchiolitis in hospitalized children: Neither frequent nor more severe. Pediatr. Pulmonol..

[37] Nunziata F., Salomone S., Catzola A., Poeta M., Pagano F., Punzi L., Lo Vecchio A., Guarino A., Bruzzese E. (2023). Clinical Presentation and Severity of SARS-CoV-2 Infection Compared to Respiratory Syncytial Virus and Other Viral Respiratory Infections in Children Less than Two Years of Age. Viruses.

[38] Flores-Pérez P., Gerig N., Cabrera-López M.I., de Unzueta-Roch J.L., Del Rosal T., Calvo C., COVID-19 Study Group in Children (2022). Acute bronchiolitis during the COVID-19 pandemic. Enfermedades Infecc. Y Microbiol. Clin..

[39] Presti S., Manti S., Gammeri C., Parisi G.F., Papale M., Leonardi S. (2024). Epidemiological shifts in bronchiolitis patterns and impact of the COVID-19: A two-season comparative study. Pediatr. Pulmonol..

[40] Dagan R., van der Beek B.A., Ben-Shimol S., Greenberg D., Shemer-Avni Y., Weinberger D.M., Danino D. (2023). The COVID-19 pandemic as an opportunity for unravelling the causative association between respiratory viruses and pneumococcus-associated disease in young children: A prospective study. eBioMedicine.

[41] Nagakumar P., Chadwick C.-L., Bush A., Gupta A. (2021). Collateral impact of COVID-19: Why should children continue to suffer?. Eur. J. Pediatr..

[42] Azar B., Hashavya S., Ohana Sarna Cahan L., Reif S., Gross I. (2023). Bronchiolitis Due to RSV and HMPV—Epidemiology, Clinical Course, and Prognosis: Experience of a Single Tertiary Center. Clin. Pediatr..

[43] Betts T.A., Darby A.E., Hussain F., Edwards M. (2024). Identifying the impact of non-pharmaceutical interventions on RSV transmission in a single-centre observational study. BMJ Paediatr. Open.

[44] Abushahin A., Toma H., Alnaimi A., Abu-Hasan M., Alneirab A., Alzoubi H., Belavendra A., Janahi I. (2024). Impact of COVID-19 pandemic restrictions and subsequent relaxation on the prevalence of respiratory virus hospitalizations in children. BMC Pediatr..

[45] Vittucci A.C., Antilici L., Russo C., Musolino A.M.C., Cristaldi S., Cutrera R., Persia S., Di Maio C.V., Raponi M., Perno C.F. (2023). Respiratory syncytial virus: Can we still believe that after pandemic bronchiolitis is not a critical issue for public health?. Eur. J. Pediatr..

[46] Nenna R., Pierangeli A., Matera L., Petrarca L., Conti M.G., Mancino E., di Mattia G., La Regina D.P., Virgili F., Papoff P. (2024). Respiratory Syncytial Virus Bronchiolitis Before and After COVID-19 Pandemic: Has the Immunity Debt Been Paid Off?. Pediatr. Infect. Dis. J..

[47] Brisca G., Strati M.F., Buratti S., Mariani M., Ferretti M., Pirlo D., Meleca V., Piccotti E., Castagnola E., Moscatelli A. (2024). The increase of bronchiolitis severity in the 2022–2023 season in an Italian tertiary children’s hospital: An isolated phenomenon or a warning sign?. Pediatr. Pulmonol..

[48] Boccard V., Prevost B., Denamur S., Peulier-Maitre E., Nathan N., Corvol H. (2024). Bronchiolitis: Increased severity in the post-COVID-19 era. Pediatr. Pulmonol..

[49] Ghirardo S., Ullmann N., Zago A., Ghezzi M., Minute M., Madini B., D‘Auria E., Basile C., Castelletti F., Chironi F. (2024). Increased bronchiolitis burden and severity after the pandemic: A national multicentric study. Ital. J. Pediatr..

[50] Muller W.J., Madhi S.A., Seoane Nuñez B., Baca Cots M., Bosheva M., Dagan R., Hammitt L.L., Llapur C.J., Novoa J.M., Saez Llorens X. (2023). Nirsevimab for Prevention of RSV in Term and Late-Preterm Infants. N. Engl. J. Med..

[51] Madhi S.A., Polack F.P., Piedra P.A., Munoz F.M., Trenholme A.A., Simões E.A.F., Swamy G.K., Agrawal S., Ahmed K., August A. (2020). Respiratory Syncytial Virus Vaccination during Pregnancy and Effects in Infants. N. Engl. J. Med..

[52] Sumsuzzman D.M., Wang Z., Langley J.M., Moghadas S.M. (2025). Real-world effectiveness of nirsevimab against respiratory syncytial virus disease in infants: A systematic review and meta-analysis. Lancet Child Adolesc. Health.

[53] Karami H., Derakhshani A., Ghasemigol M., Fereidouni M., Miri-Moghaddam E., Baradaran B., Tabrizi N.J., Najafi S., Solimando A.G., Marsh L.M. (2021). Weighted Gene Co-Expression Network Analysis Combined with Machine Learning Validation to Identify Key Modules and Hub Genes Associated with SARS-CoV-2 Infection. J. Clin. Med..

[54] Nenna R., Frassanito A., Petrarca L., Di Mattia G., Midulla F. (2020). Age Limit in Bronchiolitis Diagnosis: 6 or 12 Months?. Front. Pediatr..

