# Supplementary material for: Impact of COVID-19 Pandemic on Bronchiolitis Epidemiology in Greece
Source: Medicina (Kaunas). 2025 Sep 25;61(10):1746. doi: 10.3390/medicina61101746 (PMC12566346; doi:10.3390/medicina61101746)
Supplement: Supplementary file 1 [file medicina-61-01746-s001.zip › medicina-3837684-supplementary.pdf]

Supplementary Material

Manuscript title: *Impact of COVID-19 Pandemic on Bronchiolitis Epidemiology in Greece.*

Supplementary Table S1. Pathogenic agents detected in bronchiolitis cases, over the study periods 2017-2024.

	**2017-2018**	**2018-2019**	**2019-2020**	**2020-2021**	**2021-2022**	**2022-2023**	**2023-2024**	**2017-2024**
**Total Patients**	219	267	179	74	166	305	**295**	**1505**
**Total patients tested (for pathogen other than SARS-CoV-2) n, (%)**	213 (97,3)	262 (98,1)	175 (97,8)	68 (91,9)	163 (98,2)	303 (99,3)	**293 (99,3)**	**1477 (98,1)**
**Type of pathogen n, (%)**								
**RSV**	130 (61,0)	174 (66,4)	108 (61,7)	28 (41,2)	79 (48,5)	198 (65,3)	**202 (68,9)**	**919 (62,2)***
**SARS-CoV-2**	N/a	N/a	1 (0,6)	0	1 (0,6)	4 (1,3)	**2 (0,7)**	**8 (0,8)**
**Influenza A**	2 (0,9)	4 (1,9)	1 (0,5)	0 (0)	0 (0)	0 (0)	2 (0,7)	**9 (0,6)**
**Influenza B**	1 (0,5)	1 (0,5)	3 (1,4)	0 (0)	0 (0)	1 (0,3)	2 (0,7)	**8 (0,5)**
**Adenovirus**	1 (0,5)	0 (0)	5 (2,3)	0 (0)	1 (0,5)	7 (2,3)	12 (4,1)	**26 (1,8)***
**Parainfluenza 1**	0 (0)	0 (0)	1 (0,5)	0 (0)	0 (0)	0 (0)	1 (0,3)	**2 (0,1)**
**Parainfluenza 2**	0 (0)	0 (0)	2 (0,9)	0 (0)	1 (0,5)	1 (0,3)	0 (0)	**4 (0,3)**
**Parainfluenza 3**	1 (0,5)	3 (1,4)	0 (0)	0 (0)	0 (0)	4 (1,3)	10 (3,4)	**18 (1,2)#**
**Rhinovirus/ Enterovirus**	0 (0)	1 (0,5)	15 (7)	6 (2,8)	9 (4,2)	9 (3,0)	32 (10,9)	**72 (4,9)***
**h-MPV**	0 (0)	1 (0,5)	0 (0)	0 (0)	5 (2,3)	2 (0,7)	12 (4,1)	**20 (1,3)***
Bordetella pertussis	2 (0,9)	0 (0)	0 (0)	0 (0)	0 (0)	0 (0)	2 (0,7)	**4 (0,3)**
**Measles**	1 (0,5)	0 (0)	0 (0)	0 (0)	0 (0)	0 (0)	0 (0)	**1 (0,1)**

Mycoplasma pneumoniae	0 (0)	1 (0,5)	0 (0)	0 (0)	0 (0)	0 (0)	0 (0)	**1 (0,1)**

Abbreviations: RSV, respiratory syncytial virus; SARS-CoV-2, severe acute respiratory syndrome coronavirus 2; h-MPV, human metapneumovirus; N/a, not applicable *p<0.001 # p=0.005

Supplementary Table S2. Monthly distribution of bronchiolitis cases during the study period, 2017-2024.

**Total n, (% out of total admissions)** / **Total admissions** in PED departments	**2017-2018**	**2018-2019**	**2019-2020**	**2020-2021**	**2021-2022**	**2022-2023**	**2023-2024**	**P-value**
**November**	0 (0)	16 (3,2)	13 (2,4)	3 (1,0)	63 (19,2)	44 (7,1)	12 (2,6)	**<0.001**
	491	505	532	293	328	623	454	**<0.001**
**December**	5 (0,9)	25 (5,6)	21 (4,1)	1 (0,4)	51 (15,8)	89 (12,8)	92 (15,2)	**<0.001**
	539	446	515	280	323	695	607	**<0.001**
**January**	79 (13,6)	49 (7,7)	46 (7,0)	1 (0,3)	21 (7,1)	100 (16,0)	74 (14,9)	**<0.001**
	577	637	653	308	296	623	497	**<0.001**
**February**	46 (8,4)	63 (11)	44 (7,5)	1 (0,4)	2 (0,6)	21 (4,4)	54 (10,0)	**<0.001**
	549	571	585	276	344	478	541	**<0.001**
**March**	32 (5,8)	53 (9,8)	29 (7,8)	1 (0,4)	9 (2,7)	18 (3,2)	31 (6,6)	**<0.001**
	547	538	371	276	337	566	466	**<0.001**
**April**	18 (3,8)	22 (3,9)	7 (3,6)	2 (0,8)	7 (2,2)	1 (1,0)	19 (3,6)	**<0.001**
	476	559	192	258	314	498	533	**<0.001**
**May**	4 (0,8)	12 (2,1)	4 (1,6)	2 (0,6)	4 (1,2)	6 (1,1)	10 (2,2)	0.74

	514	566	243	334	337	525	450	**<0.001**
**June**	7 (1,5)	7 (1,4)	3 (1,0)	3 (0,9)	3 (0,7)	4 (0,8)	N/a	0.73
	478	511	304	320	444	470	449	**<0.001**
**July**	8 (1,6)	4 (0,8)	1 (0,3)	6 (2,1)	0 (0)	5 (1,0)	N/a	**0.004**
	500	522	309	285	438	497	428	**<0.001**
**August**	1 (0,2)	3 (0,7)	2 (0,8)	4 (1,7)	0 (0)	7 (1,9)	N/a	**0.005**
	409	454	245	237	417	370	389	**<0.001**
**September**	6 (1,3)	1 (0,2)	3 (1,0)	13 (4,0)	2 (0,5)	5 (1,2)	N/a	**<0.001**
	464	477	296	322	433	416	408	**<0.001**
**October**	12 (2,4)	12 (2,3)	6 (1,8)	38 (11,5)	5 (1,0)	10 (2,1)	N/a	**<0.001**
	503	514	327	329	520	475	n/a	**<0.001**

Supplementary Table S3. Number of bronchiolitis cases per age group by study period from 2017 to 2024.

Age (months) (%)	**2017-2018**	**2018-2019**	**2019-2020**	**2020-2021**	**2021-2022**	**2022-2023**	**2023-2024**	**P-value**
0-1	30 (13,7)	44 (16,5)	20 (11,2)	5 (6,8)	35 (21,1)	42 (13,7)	36 (12,2)	**0.009**
1-3	87 (39,7)	88 (33,0)	63 (35,2)	29 (39,2)	62 (37,3)	127(41,6)	107(36,3)	0.53
3-6	65 (29,7)	74 (27,7)	55 (30,7)	19 (25,8)	39 (23,5)	79 (25,9)	81 (27,5)	0.76
6-12	33 (15,1)	49 (18,3)	32 (17,9)	15 (21,1)	21 (12,7)	37 (12,1)	44 (14,9)	0.39
12-18	2 (0,9)	11 (4,1)	5 (2,8)	4 (5,4)	4 (2,4)	12 (3,9)	18 (6,1)	0.07
18-24	2 (0,9)	1 (0,4)	4 (2,2)	2 (2,7)	5 (3,0)	8 (2,6)	9 (3,1)	0.59

Supplementary Table S4. Subgroup analyses for patients ≤12 months of age at presentation. Values are represented as median (IQR).

	2017-2018	2018-2019	2019-2020	2020-2021	2021-2022	2022-2023	2023-2024	2017-2024	P-value
**Total**	**215**	**255**	**170**	**70**	**157**	**284**	**265**	**1416**	
**Admission MTS**	4 (3)	5 (3)	5 (3)	4 (2.25)	5 (2)	5 (2)	5 (3)	**5 (3)**	0.48
**Peak MTS**	5 (3)	6 (3)	5 (3)	5 (2)	5 (2)	6 (3)	6 (3)	**6 (3)**	0.27
**Requirement for respiratory support n, (%)**	191 (88.8)	219 (85.9)	138 (81.2)	53 (75.7)	117 (74.5)	238 (83.8)	223 (84.2)	**1179 (83.3)**	<0.001
**Type of respiratory support[a] n, (%)**									
**LFNO**	191	211	132	57	121	223	211	**1146**	
**HFNC**	12 (6.3)	8 (3.8)	7 (5.3)	5 (8.8)	3 (2.5)	12 (5.4)	31 (14.7)	**78 (6.8)**	<0.001
**CPAP**	5 (2.6)	1 (0.5)	1 (0.8)	0	0	1 (0.4)	0	**8 (0.7)**	0.36
**MV**	1 (0.5)	1 (0.5)	2 (1.5)	0	0	0	0	**4 (0.3)**	0.74
**Duration of respiratory support (days)**	4 (4)	4 (4)	4 (3)	4 (3)	4 (3)	4 (3)	4 (4)	**3 (3)**	0.18
**Duration of HFNC use (days)**	3 (0)	2 (0)	2 (0)	3(0)	3 (0)	4 (0)	4 (4)	**3 (0)**	**<0.001**
**Duration of hydration (days)**	3 (4)	4 (4)	3 (3)	3 (3)	3 (3)	3 (3)	4 (4)	**2 (3)**	0.36
**ICU admission n, (%)**	16 (7.7)	9 (3.6)	7 (4.1)	4 (5.7)	6 (3.8)	9 (3.2)	3 (1.1)	**54 (4.7)**	**0.04**
**Length of stay in ICU (days)**	3 (0)	3 (0)	4 (0)	2 (0)	3 (0)	4 (0)	5 (0)	**3 (0)**	0.18
**Length of Hospital Stay (days)**	4 (4)	5 (4)	4 (3)	4 (3)	4 (3)	4 (3)	5 (4)	**4 (3)**	0.49
**Hospital readmission[b] n, (%)**	1 (0.5)	7 (2.7)	4 (2.4)	1 (1.4)	1 (0.6)	6 (2.1)	3 (1.2)	**23 (2)**	0.41
**Chest X-ray performed n, (%)**	121 (56.3)	111 (43.5)	113 (66.5)	28 (40)	60 (38.2)	97 (34.2)	112 (42.3)	**642 (45.3)**	**<0.001**
**Findings in Chest X-ray n, (%)[c]**	108 (89.3)	96 (86.5)	70 (90.4)	25 (89.3)	55 (91.7)	93 (95.8)	103 (91.9)	**550 (88)**	0.37
**Pulmonary infiltrates n, (%)[c]**	18 (14.9)	23 (20.7)	18 (15.9)	6 (10)	31 (51.7)	32 (32.9)	41 (36.6)	**169 (25.1)**	**<0.001**
